# Supplementary material for: Global transcriptional analysis identifies a novel role for SOX4 in tumor-induced angiogenesis
Source: eLife. 2018 Dec 3;7:e27706. doi: 10.7554/eLife.27706 (PMC6277201; doi:10.7554/eLife.27706)
Supplement: Figure 1—source data 1. [file elife-27706-fig1-data1.docx]

| Antibody | Description | Score | Coverage | # Proteins | # Unique Peptides | # Peptides | # PSMs | Pfam IDs | # AAs | MW [kDa] | calc. pI |
| --- | --- | --- | --- | --- | --- | --- | --- | --- | --- | --- | --- |
| Diagenode | Transcription factor SOX-4 OS=Homo sapiens [SOX4_HUMAN] | 437,08 | 27,64 | 3 | 4 | 7 | 17 | Pf00505 | 474 | 47,2 | 7,36 |
| Abcam | Transcription factor SOX-4 OS=Homo sapiens [SOX4_HUMAN] | 24,90 | 9,49 | 1 | 1 | 1 | 10 | Pf00505 | 474 | 47,2 | 7,36 |
|  |  |  |  |  |  |  |  |  |  |  |  |
|  | No other SOX-family members were detected | No SOX4 detected with other antibodies or IgG control | | | |  |  |  |  |  |  |

**Figure 1 – source data 1**
