# Supplementary material for: Global transcriptional analysis identifies a novel role for SOX4 in tumor-induced angiogenesis
Source: eLife. 2018 Dec 3;7:e27706. doi: 10.7554/eLife.27706 (PMC6277201; doi:10.7554/eLife.27706)
Supplement: Figure 1—source data 2. [file elife-27706-fig1-data2.docx]

| ChIP-seq datasets | HMLE wt | HMLE-S4 untreated | HMLE-S4 treated DOX 16h | MDA-MB-231 | HCC1954 |
| --- | --- | --- | --- | --- | --- |
| SOX4 | **x** | **x** | **x** | **x** | **x** |
| H3K27ac |  | **x** | **x** |  |  |
| H3K27me3 |  | **x** | **x** |  |  |
| H3K4me3 |  | **x** | **x** |  |  |
| POL2 |  | **x** | **x** |  |  |
|  |  |  |  |  |  |
| RNA-seq datasets | **HMLE ERSOX4 untreated** | **HMLE ER** |  |  |  |
| untreated | **x** | **x** |  |  |  |
| treated | **x** | **x** |  |  |  |

**Figure 1 – source data 2**
