# Supplementary material for: Global transcriptional analysis identifies a novel role for SOX4 in tumor-induced angiogenesis
Source: eLife. 2018 Dec 3;7:e27706. doi: 10.7554/eLife.27706 (PMC6277201; doi:10.7554/eLife.27706)
Supplement: Figure 7—source data 1. [file elife-27706-fig7-data1.docx]

**Source data 1.** Correlation of nuclear SOX4 expression with clinicopathological and molecular features of invasive breast cancer

|  |  | **Nuclear SOX4 expression** | | |
| --- | --- | --- | --- | --- |
| **Feature** | **N** | **Low** | **High** | **p-value** |
|  |  | **N (%)** | **N (%)** |  |
| Histological type |  |  |  |  |
| IDC | 301 | 259 (86.0) | 42 (14.0) |  |
| ILC | 123 | 88 (71.5) | 35 (28.5) |  |
| Other | 28 | 24 (85.7) | 4 (14.3) | **0.002** |
| Histological grade |  |  |  |  |
| 1 | 80 | 77 (96.3) | 3 (3.8) |  |
| 2 | 160 | 139 (86.9) | 21 (13.1) |  |
| 3 | 195 | 139 (71.3) | 56 (28.7) | **<0.001** |
| Tumor size |  |  |  |  |
| pT1 | 202 | 177 (87.6) | 25 (12.4) |  |
| pT2 | 197 | 153 (77.7) | 44 (22.3) |  |
| pT3 | 50 | 38 (76.0) | 12 (24.0) | **0.018** |
| MAI (per 2mm^2^) |  |  |  |  |
| ≤ 12 | 230 | 210 (91.3) | 20 (8.7) |  |
| ≥ 13 | 222 | 161 (72.5) | 61 (27.5) | **<0.001** |
| Lymph node status |  |  |  |  |
| Positive | 212 | 172 (81.1) | 40 (18.9) |  |
| Negative | 220 | 182 (82.7) | 38 (17.3) | 0.667 |
| Molecular classification |  |  |  |  |
| Luminal | 377 | 311 (82.5) | 66 (17.5) |  |
| HER2-driven | 17 | 15 (88.2) | 2 (11.8) |  |
| Basal/TN | 58 | 45 (77.6) | 13 (22.4) | 0.528 |
| ERα |  |  |  |  |
| Positive | 372 | 308 (82.8) | 64 (17.2) |  |
| Negative | 80 | 63 (78.8) | 17 (21.3) | 0.392 |
| PR |  |  |  |  |
| Positive | 268 | 220 (82.1) | 48 (17.9) |  |
| Negative | 183 | 150 (82.0) | 33 (18.0) | 0.973 |
| HER2 |  |  |  |  |
| Positive | 44 | 36 (81.8) | 8 (18.2) |  |
| Negative | 407 | 334 (82.1) | 73 (17.9) | 0.968 |
