# Supplementary material for: Global transcriptional analysis identifies a novel role for SOX4 in tumor-induced angiogenesis
Source: eLife. 2018 Dec 3;7:e27706. doi: 10.7554/eLife.27706 (PMC6277201; doi:10.7554/eLife.27706)
Supplement: Figure 7—source data 2. [file elife-27706-fig7-data2.docx]

**Source data 2.** Correlation of nuclear SOX4 expression with clinicopathological features of IDC and ILC

|  | **Nuclear SOX4 expression IDC** | | |  | **Nuclear SOX4 expression ILC** | | |
| --- | --- | --- | --- | --- | --- | --- | --- |
| **Feature** | **Low** | **High** | **p-value** |  | **Low** | **High** | **p-value** |
|  | **N (%)** | **N (%)** |  |  | **N (%)** | **N (%)** |  |
| Histological grade |  |  |  |  |  |  |  |
| 1 | 50 (94.3) | 3 (5.7) |  |  | 22 (100.0) | 0 (0.0) |  |
| 2 | 86 (89.6) | 10 (10.4) |  |  | 40 (80.0) | 10 (20.0) |  |
| 3 | 113 (79.6) | 29 (20.4) | **0.013** |  | 20 (45.5) | 24 (54.5) | **<0.001** |
| Tumor size |  |  |  |  |  |  |  |
| pT1 | 134 (89.9) | 15 (10.1) |  |  | 33 (80.5) | 8 (19.5) |  |
| pT2 | 109 (82.0) | 24 (18.0) |  |  | 34 (65.4) | 18 (34.6) |  |
| pT3 | 14 (82.4) | 3 (17.6) | 0.142 |  | 20 (69.0) | 9 (31.0) | 0.265 |
| MAI (per 2mm^2^) |  |  |  |  |  |  |  |
| ≤ 12 | 129 (92.1) | 11 (7.9) |  |  | 65 (87.8) | 9 (12.2) |  |
| ≥ 13 | 130 (80.7) | 31 (19.3) | **0.004** |  | 23 (46.9) | 26 (53.1) | **<0.001** |
| Lymph node status |  |  |  |  |  |  |  |
| Positive | 125 (86.2) | 20 (13.8) |  |  | 36 (66.7) | 18 (33.3) |  |
| Negative | 124 (85.5) | 21 (14.5) | 0.866 |  | 46 (74.2) | 16 (25.8) | 0.374 |
| Molecular classification |  |  |  |  |  |  |  |
| Luminal | 207 (87.0) | 31 (13.0) |  |  | 84 (71.8) | 33 (28.2) |  |
| HER2-driven | 15 (93.8) | 1 (6.3) |  |  |  |  |  |
| Basal/TN | 37 (78.7) | 10 (21.3) | 0.216 |  | 4 (66.7) | 2 (33.3) | 0.786 |
| ERα |  |  |  |  |  |  |  |
| Positive | 206 (87.3) | 30 (12.7) |  |  | 82 (71.9) | 32 (28.1) |  |
| Negative | 53 (81.5) | 12 (18.5) | 0.236 |  | 6 (66.7) | 3 (33.3) | 0.736 |
| PR |  |  |  |  |  |  |  |
| Positive | 149 (87.6) | 21 (12.4) |  |  | 56 (69.1) | 25 (30.9) |  |
| Negative | 110 (84.0) | 21 (16.0) | 0.361 |  | 31 (75.6) | 10 (24.4) | 0.455 |
| HER2 |  |  |  |  |  |  |  |
| Positive | 34 (89.5) | 4 (10.5) |  |  | 1 (25.0) | 3 (75.0) |  |
| Negative | 225 (85.6) | 38 (14.4) | 0.514 |  | 86 (72.9) | 32 (27.1) | 0.071 |
