# Supplementary material for: Global transcriptional analysis identifies a novel role for SOX4 in tumor-induced angiogenesis
Source: eLife. 2018 Dec 3;7:e27706. doi: 10.7554/eLife.27706 (PMC6277201; doi:10.7554/eLife.27706)
Supplement: Figure 7—source data 3. [file elife-27706-fig7-data3.docx]

**Source data 3**. Clinicopathological characteristics of 452 breast cancer patients studied for the expression of SOX4.

| **Feature** | **Grouping** | **N or value** | **%** |
| --- | --- | --- | --- |
| Age (years) | Mean | 61 |  |
|  | Range | 28 to 88 |  |
|  |  |  |  |
| Histological type | IDC | 301 | 66.6 |
|  | ILC | 123 | 27.2 |
|  | Other | 28 | 6.2 |
|  |  |  |  |
| Tumor size | pT1 | 202 | 44.7 |
|  | pT2 | 197 | 43.6 |
|  | pT3 | 50 | 11.1 |
|  | Not available | 3 | 0.7 |
|  |  |  |  |
| Histological grade | 1 | 80 | 17.7 |
|  | 2 | 160 | 35.4 |
|  | 3 | 195 | 43.1 |
|  | Not available | 17 | 3.8 |
|  |  |  |  |
| MAI^#^ | ≤ 12 | 230 | 50.9 |
|  | ≥ 13 | 222 | 49.1 |
|  |  |  |  |
| Lymph node status | Negative* | 220 | 48.7 |
|  | Positive** | 212 | 46.9 |
|  | Not available | 20 | 4.4 |

^#^: per 2 mm^2^; *: negative = N0 or N0(i+); **:positive = ≥N1mi (according to TNM 7^th^ edition, 2010)
