# Supplementary material for: Global transcriptional analysis identifies a novel role for SOX4 in tumor-induced angiogenesis
Source: eLife. 2018 Dec 3;7:e27706. doi: 10.7554/eLife.27706 (PMC6277201; doi:10.7554/eLife.27706)
Supplement: Figure 7—source data 4. [file elife-27706-fig7-data4.docx]

**Source data 4**. Clinicopathological characteristics of 27 patients with metastatic breast cancer studied for the expression of SOX4.

| **Feature** | **Grouping** | **N or value** | **%** |
| --- | --- | --- | --- |
| Age (years) | Mean | 51 |  |
|  | Range | 28 to77 |  |
|  |  |  |  |
| Histological type | IDC | 22 | 81.5 |
|  | ILC | 1 | 3.7 |
|  | Other | 4 | 14.8 |
|  |  |  |  |
| Tumor size | pT1 | 7 | 25.9 |
|  | pT2 | 13 | 48.2 |
|  | pT3 | 6 | 22.2 |
|  | Not available | 1 | 3.7 |
|  |  |  |  |
| Histological grade | 1 | 0 | 0.0 |
|  | 2 | 1 | 3.7 |
|  | 3 | 26 | 96.3 |
|  |  |  |  |
| Lymph node status | Negative* | 10 | 37.0 |
|  | Positive** | 16 | 59.3 |
|  | Not available | 1 | 3.7 |
|  |  |  |  |
| ERα | Positive | 6 | 22.2 |
|  | Negative | 21 | 77.8 |
|  |  |  |  |
| PR | Positive | 4 | 14.8 |
|  | Negative | 23 | 85.2 |
|  |  |  |  |
| HER2 | Positive | 6 | 22.2 |
|  | Negative | 21 | 77.8 |
|  |  |  |  |
| Localization metastases | Brain | 17 | 63.0 |
|  | Lung | 5 | 18.5 |
|  | Skin | 4 | 14.8 |
|  | Gastro-intestinal | 1 | 3.7 |
|  |  |  |  |
| ERα metastases | Positive | 5 | 18.5 |
|  | Negative | 22 | 81.5 |
|  |  |  |  |
| PR metastases | Positive | 3 | 11.1 |
|  | Negative | 24 | 88.9 |
|  |  |  |  |
| HER2 metastases | Positive | 7 | 25.9 |
|  | Negative | 20 | 74.1 |

*: negative = N0 or N0(i+); **:positive = ≥N1mi (according to TNM 7^th^ edition, 2010)
