# Supplementary material for: Global transcriptional analysis identifies a novel role for SOX4 in tumor-induced angiogenesis
Source: eLife. 2018 Dec 3;7:e27706. doi: 10.7554/eLife.27706 (PMC6277201; doi:10.7554/eLife.27706)
Supplement: Figure 7—source data 5. [file elife-27706-fig7-data5.docx]

**Source data 5**

|  |  | **Nuclear SOX4 expression** | | |
| --- | --- | --- | --- | --- |
| **Feature** | **N** | **Low** | **High** | **p-value** |
|  |  | **N (%)** | **N (%)** |  |
|  |  |  |  |  |
| Primary tumor | 301 | 259 (86.0) | 42 (14.0) |  |
| Metastasis | 25 | 17 (68.0) | 8 (32.0) | **0.016** |
|  |  |  | | |
|  |  | **Metastasis** | | |
| **Nuclear SOX4** | **N** | **Low** | **High** | **p-value** |
|  |  | **N (%)** | **N (%)** |  |
| Primary tumor |  |  |  |  |
| Low | 15 | 11 (73.3) | 4 (26.7) |  |
| High | 8 | 2 (25.0) | 6 (75.0) | **0.039** |
